# Associations between a novel measure of sleep health and cognitive functioning in middle childhood: a crosssectional Environmental Influences on Child Health Outcomes cohort study

**DOI:** 10.1093/sleepadvances/zpaf049

**Published:** 2025-08-18

**Authors:** Joshua Marchant, Matthew Ferrell, Yingjia Wei, Kelly Baron, Courtney K Blackwell, Anat Sigal, Sarah Geiger, Susan L Schantz, Tina Hartert, Rachel S Kelly, Hooman Mirzakhani, Amy Elliott, Jody Ganiban, Dana Dabelea, Jonika Hash, Joseph B Stanford, for the ECHO Cohort Consortium, P Brian Smith, L Kristin Newby, Linda Adair, Lisa P Jacobson, Diane Catellier, Monica McGrath, Christian Douglas, Priya Duggal, Emily Knapp, Amii Kress, Courtney K Blackwell, Maxwell A Mansolf, Jin-Shei Lai, Emily Ho, David Cella, Richard Gershon, Michelle L Macy, Suman R Das, Jane E Freedman, Simon A Mallal, John A McLean, Ravi V Shah, Meghan H Shilts, Akram N Alshawabkeh, Jose F Cordero, John Meeker, Leonardo Trasande, Carlos A Camargo, Kohei Hasegawa, Zhaozhong Zhu, Ashley F Sullivan, Dana Dabelea, Wei Perng, Traci A Bekelman, Greta Wilkening, Sheryl Magzamen, Brianna F Moore, Anne P Starling, Deborah J Rinehart, Daphne Koinis Mitchell, Viren D'Sa, Sean C L Deoni, Hans-Georg Mueller, Cristiane S Duarte, Catherine Monk, Glorisa Canino, Jonathan Posner, Tenneill Murray, Claudia Lugo-Candelas, Anne L Dunlop, Patricia A Brennan, Christine Hockett, Amy Elliott, Assiamira Ferrara, Lisa A Croen, Monique M Hedderson, John Ainsworth, Leonard B Bacharier, Casper G Bendixsen, James E Gern, Diane R Gold, Tina V Hartert, Daniel J Jackson, Christine C Johnson, Christine L M Joseph, Meyer Kattan, Gurjit K Khurana Hershey, Robert F Lemanske, Susan V Lynch, Rachel L Miller, George T O'Connor, Carole Ober, Dennis Ownby, Katherine Rivera-Spoljaric, Patrick H Ryan, Christine M Seroogy, Anne Marie Singh, Robert A Wood, Edward M Zoratti, Rima Habre, Shohreh Farzan, Frank D Gilliland, Irva Hertz-Picciotto, Deborah H Bennett, Julie B Schweitzer, Rebecca J Schmidt, Janine M LaSalle, Alison E Hipwell, Catherine J Karr, Nicole R Bush, Kaja Z LeWinn, Sheela Sathyanarayana, Qi Zhao, Frances Tylavsky, Kecia N Carroll, Christine T Loftus, Leslie D Leve, Jody M Ganiban, Jenae M Neiderhiser, Scott T Weiss, Augusto A Litonjua, Cindy T McEvoy, Eliot R Spindel, Robert S Tepper, Craig J Newschaffer, Kristen Lyall, Heather E Volk, Rebecca Landa, Sally Ozonoff, Joseph Piven, Heather Hazlett, Juhi Pandey, Robert Schultz, Steven Dager, Kelly Botteron, Daniel Messinger, Wendy Stone, Jennifer Ames, Thomas G O'Connor, Richard K Miller, Emily Oken, Michele R Hacker, Tamarra James-Todd, T Michael O'Shea, Rebecca C Fry, Jean A Frazier, Rachana Singh, Caitlin Rollins, Angela Montgomery, Ruben Vaidya, Robert M Joseph, Lisa K Washburn, Semsa Gogcu, Kelly Bear, Julie V Rollins, Stephen R Hooper, Genevieve Taylor, Wesley Jackson, Amanda Thompson, Julie Daniels, Michelle Hernandez, Kun Lu, Michael Msall, Madeleine Lenski, Rawad Obeid, Steven L Pastyrnak, Elizabeth Jensen, Christina Sakai, Hudson Santos, Jean M Kerver, Nigel Paneth, Charles J Barone, Michael R Elliott, Douglas M Ruden, Chris Fussman, Julie B Herbstman, Amy Margolis, Susan L Schantz, Sarah Dee Geiger, Andrea Aguiar, Karen Tabb, Rita Strakovsky, Tracey Woodruff, Rachel Morello-Frosch, Amy Padula, Joseph B Stanford, Christina A Porucznik, Angelo P Giardino, Rosalind J Wright, Robert O Wright, Brent Collett, Nicole Baumann-Blackmore, Ronald Gangnon, Daniel J Jackson, Chris G McKennan, Jo Wilson, Matt Altman, Judy L Aschner, Annemarie Stroustrup, Stephanie L Merhar, Paul E Moore, Gloria S Pryhuber, Mark Hudak, Ann Marie Reynolds Lyndaker, Andrea L Lampland, Burton Rochelson, Sophia Jan, Matthew J Blitz, Michelle W Katzow, Zenobia Brown, Codruta Chiuzan, Timothy Rafael, Dawnette Lewis, Natalie Meirowitz, Brenda Poindexter, Tebeb Gebretsadik, Sarah Osmundson, Jennifer K Straughen, Amy Eapen, Andrea Cassidy-Bushrow, Ganesa Wegienka, Alex Sitarik, Kim Woodcroft, Audrey Urquhart, Albert Levin, Tisa Johnson-Hooper, Brent Davidson, Tengfei Ma, Emily S Barrett, Martin J Blaser, Maria Gloria Dominguez-Bello, Daniel B Horton, Manuel Jimenez, Todd Rosen, Kristy Palomares, Lyndsay A Avalos, Yeyi Zhu, Kelly J Hunt, Roger B Newman, Michael S Bloom, Mallory H Alkis, James R Roberts, Sunni L Mumford, Heather H Burris, Sara B DeMauro, Lynn M Yee, Aaron Hamvas, Antonia F Olidipo, Andrew S Haddad, Lisa R Eiland, Nicole T Spillane, Kirin N Suri, Stephanie A Fisher, Jeffrey A Goldstein, Leena B Mithal, Raye-Ann O DeRegnier, Nathalie L Maitre, Ruby H N Nguyen, Meghan M JaKa, Abbey C Sidebottom, Michael J Paidas, JoNell E Potter, Natale Ruby, Lunthita Duthely, Arumugam Jayakumar, Karen Young, Isabel Maldonado, Meghan Miller, Jonathan L Slaughter, Sarah A Keim, Courtney D Lynch, Kartik K Venkatesh, Kristina W Whitworth, Elaine Symanski, Thomas F Northrup, Hector Mendez-Figueroa, Ricardo A Mosquera, Margaret R Karagas, Juliette C Madan, Debra M MacKenzie, Johnnye L Lewis, Brandon J Rennie, Bennett L Leventhal, Young Shin Kim, Somer Bishop, Sara S Nozadi, Li Luo, Barry M Lester, Carmen J Marsit, Todd Everson, Cynthia M Loncar, Elisabeth C McGowan, Stephen J Sheinkopf, Brian S Carter, Jennifer Check, Jennifer B Helderman, Charles R Neal, Lynne M Smith

**Affiliations:** Department of Pediatrics, University of Utah, Salt Lake City, UT, United States; Department of Psychology, Brigham Young University, Provo, UT, United States; Department of Pediatrics, University of Utah, Salt Lake City, UT, United States; Department of Pediatrics, University of Utah, Salt Lake City, UT, United States; Department of Family and Preventive Medicine, University of Utah, Salt Lake City, UT, United States; Feinberg School of Medicine, Northwestern University, Evanston, IL, United States; Department of Pediatrics, Hackensack University Medical Center, Hackensack, NJ, United States; Community Health Program and Beckman Institute, University of Illinois Urbana-Champaign, Champaign, IL, United States; Comparative Biosciences Program and Beckman Institute, University of Illinois Urbana-Champaign, Champaign, IL, United States; Department of Pediatrics, Vanderbilt University School of Medicine, Nashville, TN, United States; Channing Division of Network Medicine, Department of Medicine, Harvard Medical School and Brigham and Women’s Hospital, Boston, MA, United States; Channing Division of Network Medicine, Department of Medicine, Harvard Medical School and Brigham and Women’s Hospital, Boston, MA, United States; Department of Pediatrics, Avera Research Institute, Sioux Falls, SD, United States; Department of Psychological and Behavioral Sciences, George Washington University, Washington, DC, United States; Lifecourse Epidemiology of Adiposity and Diabetes (LEAD) Center, Colorado School of Public Health, Aurora, CO, United States; Department of Child, Family, and Population Health, University of Washington, Seattle, WA, United States; Department of Family and Preventive Medicine, University of Utah, Salt Lake City, UT, United States

**Keywords:** sleep health, middle childhood, cognitive functioning, inhibitory control, working memory

## Abstract

**Study Objectives:**

Research linking children’s sleep to cognitive outcomes is inconsistent and has largely focused on one aspect of sleep, such as duration, rather than measuring multiple dimensions of sleep health. We hypothesized that children’s sleep health would be positively associated with inhibitory control and cognitive functioning.

**Method:**

We cross-sectionally assessed 1595 participants (ages 7–11) from the Environmental influences on Child Health Outcomes cohort using the NIH Toolbox Cognition Battery, Environmental influences on Child Health Outcomes Sleep Health of Children and Adolescents questionnaire, and Patient Reported Outcome Measurement Information System Sleep Disturbance/Sleep-related Impairment instruments. We created a novel scale measuring sleep health using dichotomous “good–bad” cutoffs for sleep duration, timing, latency, satisfaction, and alertness. We used generalized estimating equations and random forest models to examine associations between sleep health and inhibitory control, working memory, processing speed, cognitive flexibility, episodic memory, reading decoding, and receptive vocabulary.

**Results:**

Sleep health did not have statistically significant associations with any aspect of cognitive functioning. Notably, over 75 per cent of our sample had good sleep health.

**Conclusions:**

This study assessed sleep health as a multi-faceted construct, distinguishing between “good” and “poor” sleep health across several domains. The absence of statistically significant associations between sleep health and cognitive functioning suggests children’s cognitive functioning may not be cross-sectionally related to multidimensional sleep health measures. Experimentally manipulating key sleep domains such as duration or timing (as done in prior research) may be more robust. Future research might benefit from examining the cumulative impact of poor sleep health over time.

**Statement of significance**

It is essential to identify variables affecting children’s cognitive functioning because it is associated with many important health outcomes. This is the first study, to our knowledge, that captures a multidimensional measure of sleep health and cognitive functioning for children aged 7–11 years, providing results generalizable to a broad range of children, not just those with a cognitive impairment or sleep disorder. By measuring sleep health from a multidimensional standpoint, we avoid the pitfall of simply measuring sleep as the presence/non-presence of a sleep disorder. Our study findings indicate that subjectively measured sleep health is not associated with cognitive functioning in a large, nationally representative sample. Future studies would benefit from measuring sleep health longitudinally, building on our retrospective, cross-sectional study.

## Introduction

Healthy sleep in childhood is critical to many aspects of cognitive, emotional, and physical well-being. [1–5] Prior research suggests that sleep deprivation can impair inhibitory control and increase emotional reactivity, which may contribute to negative mood states and impulsivity in sleep-deprived individuals [6]. The most prominent theory to explain this association is the synaptic homeostasis theory, which posits that the brain downscales synaptic connections during sleep, making the brain more efficient at performing cognitive functions [7]. Cognitive functioning during child development refers to a broad set of mental processes involved in acquiring knowledge, reasoning, problem solving, and paying attention [8].

Although the associations between children’s sleep and cognitive functioning have been extensively studied, results are still mixed in terms of individual cognitive outcomes and how sleep is measured [2, 9–11]. For example, Astill *et al.* [1] found in a meta-analysis of 86 studies involving 35 936 children that reduced sleep duration was linked to lower overall cognitive functioning. Astill *et al.* [1] also noted that decreased sleep duration was associated with impaired executive functioning, which refers to the mental processes regulated by the prefrontal cortex that support goal-directed behavior, including inhibitory control, working memory, and cognitive flexibility. Conversely, Short *et al.* [9] found in a meta-analysis of 19 studies using only objectively measured sleep duration that children’s sleep was associated with overall cognition but not with executive functioning.

Some individual studies have found small-to-medium associations between children’s sleep and inhibitory control, working memory, and cognitive flexibility, while other studies have found no associations between these factors [12–19]. Similarly, other studies have shown inconsistent associations between children’s sleep and processing speed [2, 11, 20–23]. Children’s sleep is also highly associated with memory consolidation [20, 21], but few studies have examined how sleep affects the ability to encode new memories. The results of various studies suggest that children’s sleep may not affect memory encoding at all [1, 9, 19, 22]. The impacts of children’s sleep on language development have not been studied extensively, but the few available studies have demonstrated some associations [2, 9, 23, 24].

While there is a trend in findings of the associations of sleep with overall cognition in childhood, there is a need for research to examine how children’s sleep health is associated with individual cognitive domains. While much of the prior literature has focused on the effects of extreme sleep deprivation or pathology on cognitive functioning, growing evidence suggests that even subtle disruptions across multiple sleep domains could contribute to differences in health outcomes [11, 25].

In general, prior research on children’s sleep and cognition has been limited by including only one domain of sleep (i.e. duration) rather than measuring sleep multidimensionally. The studies cited above have found various associations between sleep duration, timing, daytime alertness, and cognitive functioning, but none examined combined measures of these variables, such as sleep health. Pediatric sleep health is represented by Meltzer *et al.* [26] using a holistic, six-factor model that includes sleep-related behaviors, satisfaction with sleep, alertness during waking hours, appropriate sleep timing, sleep efficiency, and adequate sleep duration for age. Whereas sleep has often been evaluated in research as including only one domain (duration), sleep health is a multi-faceted construct that refers to optimal sleep characteristics and emphasizes the positive role of sleep for overall health [27]. In contrast, sleep problems typically refer to clinically significant disturbances such as insomnia, sleep apnea, or chronic sleep deprivation.

Many of the prior studies on children’s sleep and cognition have also been limited by small sample sizes, highlighting the need for studies with larger participant pools. No studies to our knowledge have tested a cross-sectional relationship between a multidimensional model of children’s sleep health and cognitive functioning. A large-scale, cross-sectional design is the first step in establishing correlations that can be further studied in longitudinal and experimental research.

In the present study, we first aimed to determine whether children’s sleep health was associated with inhibitory control in a large sample of children. After our first round of results demonstrated no associations, we conducted additional post-hoc analyses examining whether sleep health was associated with other aspects of cognitive functioning. We specifically examined five domains of sleep health in middle childhood (sleep duration, timing/chronotype, satisfaction/quality, daytime alertness, and latency). We did not include sleep behaviors from the pediatric sleep health model due to a lack of relevant data in our questionnaires. We used sleep onset latency (i.e. time it takes to fall asleep after getting in bed) as a proxy variable for sleep efficiency due to the data available in our dataset. We hypothesized that (a) higher sleep health composite scores in middle childhood would be associated with better inhibitory control and cognitive functioning; (b) a composite score of pediatric sleep health would be the most important variable in our model; and (c) based on previous literature demonstrating associations among sleep duration, timing, and cognitive functioning, the individual dimensions that would have the greatest predictive power in our sleep health model would be sleep duration and sleep timing.

## Methods

### Study design and population

**Figure 1:**
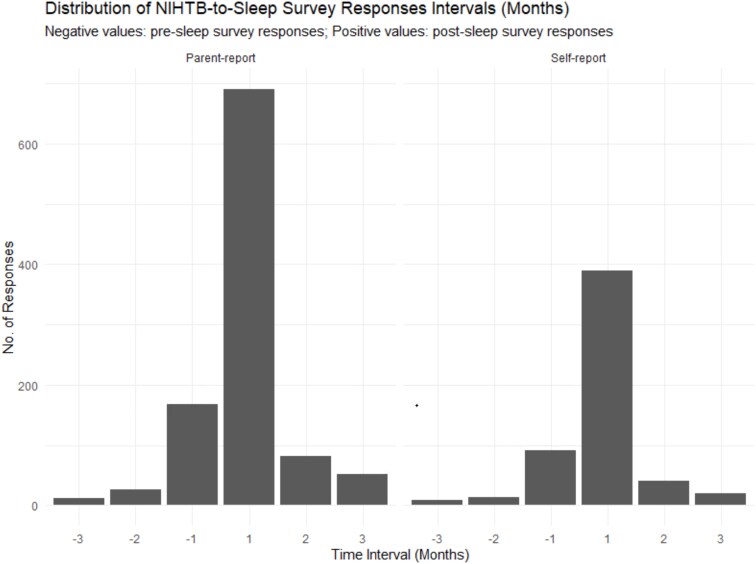
Distribution of NIH Toolbox (NIHTB) to sleep survey response collection time intervals (in months). NIHTB indicates National Institutes of Health Toolbox.

**Figure 2:**
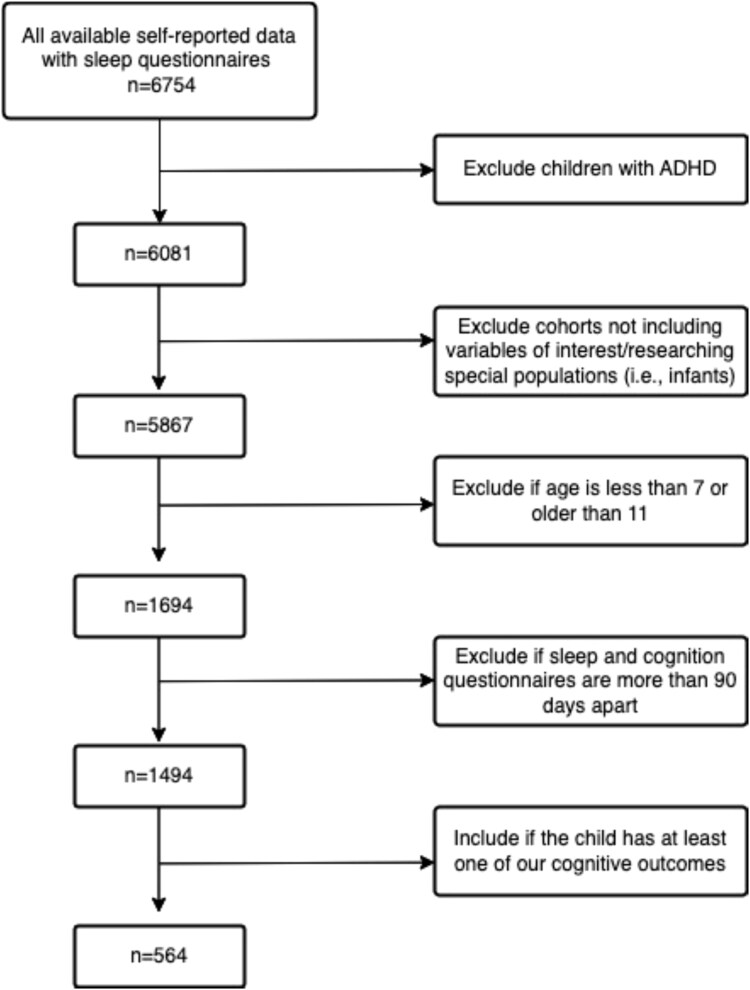
Flowchart of inclusion/exclusion criteria (self-report). ADHD indicates attention-deficit/hyperactivity disorder.

**Figure 3:**
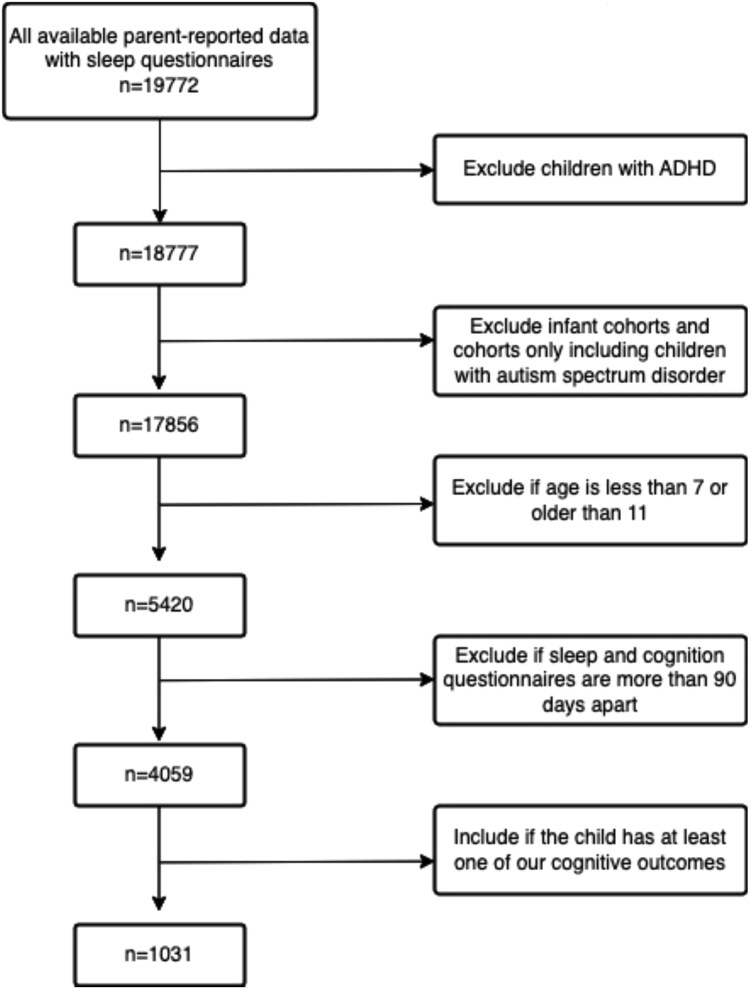
Flowchart of inclusion/exclusion criteria (parent report). ADHD indicates attention-deficit/hyperactivity disorder.

Our study used data from the National Institutes of Health (NIH)-funded Environmental Influences on Child Health Outcomes (ECHO) cohort to cross-sectionally assess relations between sleep health and cognitive functioning. The ECHO Cohort is a consortium of 69 established pediatric cohorts in the United States designed to address research questions about the effects of a broad range of early environmental exposures on child health and development [28]. Participants were eligible for inclusion in the current study if they or a parent had completed the ECHO Sleep Health of Children and Adolescents survey, Patient Reported Outcome Measurement Information System® (PROMIS) Sleep Disturbance and Sleep-related Impairments questionnaires, and at least one NIH Toolbox inhibitory control task in middle childhood (ages 6–11 years). Children were excluded if they had a diagnosis of autism spectrum disorder, as explorations of autism spectrum disorder and inhibitory control were beyond the scope of the present investigation. We also excluded participants if they had measurements of sleep health and cognition that were more than 90 days apart [29], although the temporal order of sleep health and cognitive functioning was not considered. Over 70 per cent of sleep questionnaires and cognitive variables were collected within 1 month of each other, and most sleep responses were collected before cognitive measures (see Figure 1). For children with proxy sleep data from multiple sources, we prioritized surveys completed by the mother. The final sample consisted of 1595 participants from 23 cohorts (see Figures 2 and 3 for a flowchart of inclusion and exclusion criteria). Data were collected between August 16, 2017, and February 20, 2023, via the ECHO common protocol. Participant recruitment occurred by email, telephone, or text, and assessments and surveys were completed either at in-person visits or remotely. Single and cohort-specific institutional review boards monitored human subject activities, and data were collected in the centralized ECHO Data Analysis Center. All participants provided written informed consent.

### Measurements

#### Sleep health

Sleep health was assessed using the ECHO Sleep Health of Children and Adolescents questionnaire (specifically using the questions asking what time children fall asleep, when they wake up, and how long it takes them to fall asleep to determine duration, timing, and sleep latency) and the PROMIS sleep-related impairment and sleep disturbance questionnaires (for satisfaction and alertness) [30]. As stated above, we used sleep onset latency to estimate efficiency. Timing in our measures referred to the average sleep midpoint (based on large, nationally representative samples [31]) rather than consistency from night to night. Because the ECHO Sleep Health questionnaire is composed of a combination of PROMIS measures, psychometric properties for the PROMIS measures are reported here. The PROMIS measures have been well-validated using sleep actigraphy [30], are calibrated and normed to the general United States population, and use a standardized t-score metric (*M* = 50, *SD* = 10). The PROMIS parent-report scales (Sleep-related impairment and sleep disturbance) demonstrated good internal consistency in our sample ($\alpha$ = 0.86 for both scales). The self-reported Sleep-related Impairment and Sleep Disturbance scales had adequate internal consistency in our sample ($\alpha$ = 0.79 and $\alpha$ = 0.65, respectively). These measures were administered to parents and children via an online platform (Research Electronic Data Capture [REDCap]). Child self-reporting was used for children aged either 8 or 9 years and older (depending on cohort-specific guidelines), with parent reports generally used for younger children. Sleep health dimensions measured by these questionnaires included nocturnal sleep duration, timing/chronotype, satisfaction/quality, alertness, and latency.

We created a composite score of sleep health by changing each element of sleep health (duration, timing/chronotype, satisfaction/quality, alertness, and latency) into a dichotomous variable. Rather than relying on assumptions about linear relationships (e.g. that each extra minute of sleep is equally beneficial), dichotomizing each sleep health domain allowed for clearer differentiation between children with “poor” vs. “good” sleep health in a way that aligns with clinical sleep guidelines. While dichotomizing reduces variability, it avoids overfitting. Dichotomizing each sleep domain to create a composite score is also consistent with prior research [32, 33]. Each dimension of sleep health was scored as either 0 (poor sleep health) or 1 (healthy sleep). The cutoffs for these scores were determined by (1) the PROMIS t-scores for sleep satisfaction/quality and alertness, (2) guidance from the American Academy of Pediatrics and National Sleep Foundation for sleep duration, and (3) prior literature examining mean sleep midpoints and sleep latency in large samples of children for sleep timing and latency (Table 1 [31, 34, 35]). This scoring procedure resulted in a composite score of sleep health ranging from 0 to 5. We did not change cutoffs depending on age in our sample because sleep recommendations are roughly similar for children aged 7–11 years [34].

**Table 1:** Sleep health composite scoring

Sleep health domain	Definition	Good sleep health, % (*n*)	Poor sleep health, % (*n*)	Missing data, % (*n*)
Parent-report				
Duration [34]	Good: 9–11 hours; Poor: <9 hours; >11 hours	74% (762)	26% (269)	0.05% (5)
Timing [31]	Good: Sleep midpoint standard deviation between 1:00 and 3:00; Poor: Midpoint <1:00 or > 3:00	78% (736)	22% (295)	8.9% (92)
Latency [35]	Good: Sleep latency <25 minutes; Poor: Sleep latency ≥25 minutes	58% (585)	42% (446)	1.5% (15)
Satisfaction/quality	Good: t-score < 60; Poor: t-score ≥ 60	85% (481)	15% (550)	45.0% (464)
Alertness	Good: t-score < 60; Poor: t-score ≥ 60	90% (523)	10% (508)	43.7% (450)
Self-report				
Duration [34]	Good: 9–11 hours; Poor: <9 hours; >11 hours	77% (435)	23% (129)	0% (0)
Timing [31]	Good: Sleep midpoint standard deviation between 1:00 and 3:00; Poor: Midpoint <1:00 or > 3:00	78% (439)	22% (125)	0% (0)
Latency [35]	Good: Sleep latency <25 minutes; Poor: Sleep latency ≥ 25 minutes	61 % (342)	39% (222)	0% (0)
Satisfaction/quality	Good: t-score < 60; Poor: t-score ≥ 60	78% (312)	22% (252)	29.9% (139)
Alertness	Good: t-score < 60; Poor: t-score ≥ 60	84% (267)	16% (297)	44.0% (204)

#### NIH toolbox cognition battery

The NIH Toolbox Cognition Battery was developed as part of the NIH Blueprint for Neuroscience Research to provide a brief, standardized assessment of neurocognitive function [36]. The Cognition Battery is designed to measure child and adult cognition quickly and reliably across multiple developmental stages. Assessments of inhibitory control, working memory, cognitive flexibility, processing speed, episodic memory, receptive vocabulary, and reading decoding ability are included in the battery. Raw scores are transformed into standard scores (*M* = 100, *SD* = 15). Reliability and convergent/discriminant validity for these scales range from adequate to excellent (more information about reliability, validity, background, and scoring procedures can be found in Weintraub *et al.* [36]).

### Analytic plan

We used an unstructured correlation generalized estimating equation (GEE) model, clustering by cohort. This approach allowed arbitrary correlations between the same cluster observations. To avoid the potential issue of false positives created by running numerous analyses (14 total GEE analyses), we set our critical alpha value to 0.01. All analyses were performed using R 4.2.2 (R Studio, Vienna, Austria) and SQL Server Management Studio 18.12.1 (Microsoft Corporation, Redmond, WA).

In our first primary analysis, we used GEEs to assess the association between the sleep health composite score and children’s inhibitory control. When we discovered a null relationship between children’s sleep health and inhibitory control, we expanded the analysis to include working memory, cognitive flexibility, processing speed, episodic memory, receptive vocabulary, and reading decoding. After consultation with experts in sleep science, we included child sex, child ethnicity, child race, child birth weight, and family socioeconomic status (using the Organization for Economic Co-operation and Development income cutoffs) as potential confounding variables that might influence sleep and/or cognitive functioning. We did not include age as a covariate because (1) we were using age-corrected t-scores and (2) guidelines for sleep for children aged 7–11 years are roughly the same across that developmental period [34].

In our second primary analysis, we used a random forest model to determine which category of children’s sleep health (duration, timing/chronotype, satisfaction/quality, alertness, or latency) had the most predictive power in the model examining associations between sleep health cognitive functioning (using a cognitive functioning composite score calculated by averaging t-scores from the NIH Toolbox). Predictive power was indicated by an increase in the mean standard error when those covariates were removed from the model. For our random forest model, we used continuous sleep variables instead of the dichotomized scores used for the sleep health composite. This random forest procedure has been used in previous literature examining sleep health from a multidimensional standpoint [25]. Because there are often discrepancies between parent-report and child self-report sleep data [37, 38], we ran separate parallel primary analyses for parent- and self-report data.

## Results

### Descriptive analyses

We had a large sample size (*n* = 1031 for parent report, *n* = 564 for self-report; data pulled from 23 cohorts), but a significant amount of data was missing for some of the variables (Tables 1 and 2). For example, over 40 per cent of the data were missing for sleep alertness and sleep satisfaction. We used multiple imputations to account for these missing variables under the assumption that the data were missing at random. This assumption was based on the following arguments: (1) Cohorts collected sleep/cognitive measures at varying periods, and if one cognitive or sleep measure was completed more than 3 months apart from the other measures, it could not be included in the analysis; (2) some cognitive measures were collected remotely during the COVID-19 pandemic, making it impossible to use the processing speed measure; and (3) children under age 8 were not tested for reading decoding or working memory (parent-report data were generally used for children under age 9, which is one reason for missing reading decoding scores). We ran a sensitivity analysis to determine whether the imputation had significant effects (Tables S1 and S2). The results of the sensitivity analysis indicated that the multiple imputation did not significantly alter the results.

**Table 2:** Descriptive statistics for demographic and cognitive variables

	*N* (%)	*M*	*SD*	% Missing
Total sample	1595	—	—	—
Parent-report sample	1031	—	—	—
Self-report sample	564	—	—	—
**Demographic characteristics (total sample)**				
Age	—	8.8	1.85	—
Sex				
Female	504 (49)	—	—	—
Male	527 (51)	—	—	—
Race				
White	536 (53)	—	—	—
African American	265 (26)	—	—	—
Other race	215 (21)	—	—	—
Ethnicity				
Hispanic	188 (18)	—	—	—
Not Hispanic	843 (82)	—	—	—
OECD Income				
<$30 000	344 (39)	—	—	—
$30 000–$49 999	449 (50)	—	—	—
$50 000–$74 999	53 (6)	—	—	—
>$75 000	47 (5)	—	—	—
Gestational age at birth				
<32 weeks	244 (25)	—	—	—
32–37 weeks	65 (7)	—	—	—
≥37 weeks	653 (68)	—	—	—
**Cognitive outcomes (self-report sample)**				
Inhibitory control	556	96.02	13.29	1.42%
Working memory	553	100.94	15.6	1.95%
Processing speed	552	91.74	20.67	2.13%
Cognitive flexibility	552	98.09	14.06	2.13%
Episodic memory	554	101.96	14.86	1.77%
Reading decoding	539	101.55	15.93	4.43%
Receptive vocabulary	552	106.06	15.31	2.13%
**Cognitive outcomes (parent-report sample)**				
Inhibitory control	969	94.86	14.4	6.01%
Working memory	599	97.28	16.43	41.90%
Processing speed	586	89.38	20.74	43.16%
Cognitive flexibility	804	93.12	14.48	22.02%
Episodic memory	804	99.79	17.52	22.02%
Reading decoding	562	98.64	17.22	45.49%
Receptive vocabulary	974	101.28	18.11	5.53%

OECD indicates Organization for Economic Co-operation and Development.

Table 1 also demonstrates that most children (over 75 per cent) in our sample had adequate sleep duration, timing, satisfaction with sleep, and alertness during the day. Sleep latency scores were closer to 60 per cent, with many children falling asleep within 20 minutes.

### Analysis of sleep, inhibitory control, and cognitive functioning

We hypothesized that better sleep health in middle childhood would be associated with better inhibitory control and cognitive functioning (hypothesis a). We did not find any significant associations between our composite score of sleep health and inhibitory control, working memory, cognitive flexibility, episodic memory, reading decoding, or receptive vocabulary (Tables 3 and 4). The results were nonsignificant (*p* < .01) for both self-report and parent-report data. Higher sleep health appeared to be associated with lower inhibitory control in the self-report sample ($b$ = -0.98 [-1.68, -0.27], *p* = .02) and with higher receptive vocabulary in the parent-report sample ($b$ = 0.87 [0.22, 1.52], *p* = .02). However, these associations were not statistically significant based on our alpha level of 0.01, and using standardized $\beta$ values clearly shows that these associations were minuscule. A 1-unit increase in the sleep health composite score was associated with a -0.07 *SD* difference in inhibitory control (in the self-report sample) and a 0.05 *SD* difference in receptive vocabulary (in the parent-report sample). Confidence intervals suggested that these associations could range from small to almost non-existent.

**Table 3:** General estimating equation results evaluating associations between self-reported sleep health and cognitive variables

Primary outcome	Unadjusted coefficient	Adjusted coefficient^*^	SE	95% CI LL	95% CI UL	*P*	Standardized $\boldsymbol{\beta}$ ($\boldsymbol{b}/\boldsymbol{SD}\Big)$
Sleep health composite and inhibitory control	−0.98	−0.98	0.43	−1.68	−0.27	.02	0.002
Sleep health composite and working memory	0.11	0.07	0.57	−0.83	1.04	.85	−0.073
Sleep health composite and processing speed	−0.2	−0.21	0.74	−1.43	1.03	.79	0.007
Sleep health composite and cognitive flexibility	0.02	0.04	0.49	−0.78	0.83	.96	−0.001
Sleep health composite and episodic memory	0.2	0.19	0.49	−0.61	1.01	.68	0.013
Sleep health composite and reading decoding	0.74	0.73	0.54	−0.15	1.63	.17	0.046
Sleep health composite and receptive vocabulary	0.55	0.54	0.46	−0.21	1.32	.23	0.036

CI indicates confidence interval, LL, lower limit, SD, standard deviation, SE, standard error, UL, upper limit.

^*^Adjusted for race, annual income, birth weight, and sex.

**Table 4:** General estimating equation results evaluating associations between parent-reported sleep health and cognitive variables

Primary Outcome	Unadjusted coefficient	Adjusted coefficient^*^	SE	95% CI LL	95% CI UL	*P*	Standardized $\boldsymbol{\beta}$ ($\boldsymbol{\beta} /\boldsymbol{SD}\Big)$
Sleep health composite and inhibitory control	0.54	0.55	0.41	−.026	1.1	.31	0.029
Sleep health composite and working memory	0.28	0.3	0.51	−0.57	1.13	.58	0.037
Sleep health composite and processing speed	0.53	0.54	0.69	−0.61	1.67	.44	0.017
Sleep health composite and cognitive flexibility	0.42	0.43	0.41	−0.26	1.1	.31	0.025
Sleep health composite and episodic memory	0.89	0.89	0.48	0.11	1.67	.06	0.051
Sleep health composite and reading decoding	0.34	0.37	0.48	−0.45	1.13	47	0.020
Sleep health composite and receptive vocabulary	0.87	0.89	0.4	0.22	1.52	.03	0.048

CI indicates confidence interval, LL, lower limit, SD, standard deviation, SE, standard error, UL, upper limit.

^*^Adjusted for race, annual income, birth weight, and sex.

### Analysis of the random Forest map of variable importance

**Figure 4:**
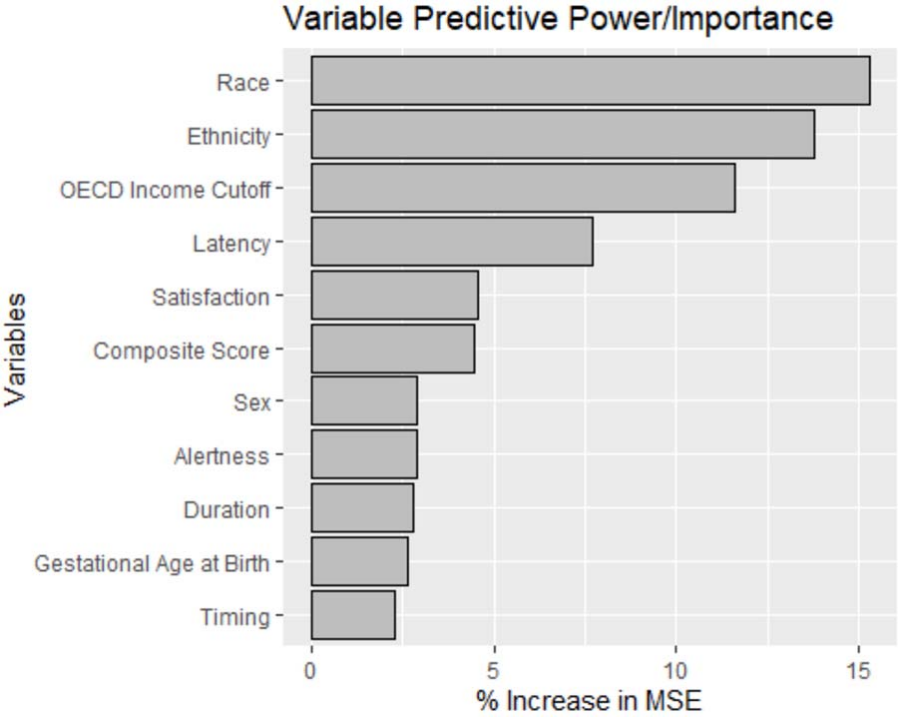
Variable importance map for each domain of sleep health and covariates with the composite cognitive score as the outcome variable for the self-report sample. MSE indicates mean standard error; OECD, Organization for Economic Co-operation and Development.

**Figure 5:**
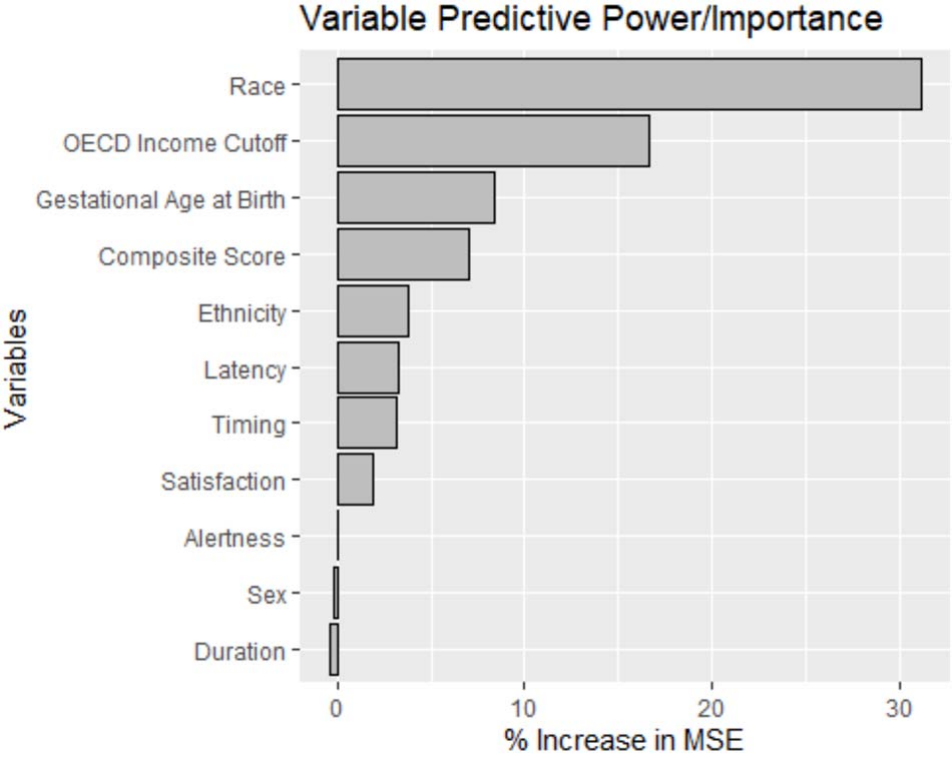
Variable importance map for each domain of sleep health and covariates with the composite cognitive score as the outcome variance for the parent-report sample. MSE indicates mean standard error; OECD, Organization for Economic Co-operation and Development.

We hypothesized that the composite score of pediatric sleep health would be the most predictive variable in our sleep health model (hypothesis b) and that, examined individually, the most predictive dimensions of sleep health would be sleep duration and sleep timing (hypothesis c). We found that the random forest models demonstrated a good fit for our variables of interest and cognitive scores (Figures S1 and S2), suggesting that the variable importance maps for the individual domains of sleep health and covariates were accurate. However, in the parent-report data, 50.02 per cent of participants were missing the cognitive composite score, making the variable importance map for parent-report data less accurate. In both models, covariates such as child race and family socioeconomic status had more predictive power than the sleep variables (Figures 4 and 5). Thus, hypothesis b (that the sleep composite would be the most predictive variable) was not supported by the data. The sleep composite score was more important in the model than the individual dimensions of sleep health (indicated by a ~7 per cent increase in the mean standard error when excluding the composite from the model) for the parent-report but not the self-report sample, providing some support for hypothesis c (that the most important dimensions of sleep health with cognitive functioning would be duration and timing).

## Discussion

In the present study, we built on previous literature examining children’s sleep and inhibitory control by measuring sleep from a multidimensional standpoint with a large sample from the ECHO-wide cohort. Importantly, this is the first study we are aware of that examines cross-sectional relationships between a multi-faceted measure of sleep health and cognitive functioning in middle childhood.

Somewhat surprisingly, we found no statistically significant associations between children’s sleep health and inhibitory control or between children’s sleep health and any other cognitive variable in the study. For the self-report sample, the association of sleep health with inhibitory control approached significance, indicating that better sleep health was associated with slightly worse inhibitory control. For the parent-report sample, the association with receptive vocabulary approached significance, indicating that better sleep health was associated with slightly better receptive vocabulary. These variables had very small associations with sleep health and appeared in only one sample (parent report or self-report). In addition, the finding that better sleep health was associated with worse inhibitory control is counterintuitive. Although these associations could be possible, we find the assumption that they were spurious and resulted from multiple comparisons to be more plausible. We also found that the majority of children in our sample had adequate sleep duration, timing, satisfaction with sleep, and alertness during the day.

On one hand, our results are unexpected given prior findings indicating significant associations between sleep and executive functioning [1, 12, 16]. On the other hand, our results are in line with numerous other studies that failed to find these same associations [9, 13, 14]. These inconsistencies in prior research suggest that while sleep may play a role in cognitive functioning, its effects are likely small and influenced by multiple interacting factors.

Notably, our findings suggest that sleep health may have small associations with receptive vocabulary (in the parent-report sample). This offers some support for previous studies [9, 23]. Verbal intelligence is sometimes used as a proxy for overall intelligence, highlighting the importance of this finding [39]. However, since this association was not observed in the self-report sample, its reliability should be interpreted with caution.

Generally, the studies that conflict most with our findings showing no association between sleep and cognitive functioning used an experimental design to either restrict or extend sleep over multiple days [17–19, 40]. The literature generally demonstrates that cumulative sleep loss over time is most robustly predictive of cognitive functioning. In contrast, studies that restrict sleep over one night or that measure sleep cross-sectionally have less associative power, although this is not always the case [9, 13, 22, 23, 41].

Interestingly, even though we did not find statistically significant associations between children’s sleep health and inhibitory control, we did find that our sleep health composite score was a more important variable in our random forest model than other sleep variables for parent-reported sleep. These results should be interpreted with caution, as the random forest variable importance map determines only which variables reduce the model’s mean standard error, not necessarily how highly associated the variable is with cognitive functioning. In addition, other covariates had more importance than sleep in the random forest model, suggesting potential disparities in education and healthcare experienced among disadvantaged groups [42]. This suggests that while sleep health may contribute to cognitive development, it is likely only one piece of a broader set of influences. In practice, improvements in sleep health alone may not drastically alter cognitive outcomes.

Overall, research linking children’s sleep to cognitive functioning is inconclusive, with findings varying depending on the methodology and measurement approaches. Recognizing that variations in sleep health within a normative range may not have substantial effects on cognitive functioning, we explicitly differentiated between children with “poor” and “good” sleep health by dichotomizing each sleep health dimension and creating a composite score to capture the combined impact of multiple sleep disruptions. Our results did not reveal strong associations, suggesting no association between sleep health and cognitive functioning when measured at a single time point. However, this does not necessarily mean that sleep health is unrelated to cognitive functioning. For example, prior research suggests that the cumulative burden of sleep deprivation over time could be more predictive of cognitive outcomes. Accordingly, disruptions in various sleep domains (such as duration) may have more impact when measured longitudinally than cross-sectionally. Prior research suggests the effect of sleep on children’s cognitive functioning is small and impacted by complex behavioral and environmental factors, such that tightly controlled experimental designs with multi-night sleep restriction are necessary to detect associations. Moderating variables used in other studies may also influence the variable results in the research literature.

### Limitations

One limitation of our study is the use of retrospective parent- and self-reported children’s sleep health. Most research indicates that self-reported sleep is only moderately correlated with actual sleep variables, with parent reports being even less accurate [37, 43]. Another limitation is that with our cross-sectional design, we could not detect causality between any of our exposures and outcomes. We also collected sleep and cognitive measures in this study at different time points, assuming that children’s sleep health would remain relatively constant over 3 months. We mitigated this issue by excluding participants whose data were collected more than 90 days apart. While respondents tend to generalize answers to PROMIS surveys, regardless of the time frame they are supposed to be thinking about [44], it is possible that the sleep questionnaires represented a particular week of sleep rather than being representative of children’s overall sleep patterns. While this is a limitation, most participants responded to sleep questionnaires within a month of cognitive measures, and the majority answered sleep questionnaires before completing cognitive testing. Additionally, we did not include environmental variables related to sleep, such as air quality or noise levels.

Another potential limitation is that we lost the power of continuous variables by converting each of the sleep health domains into a dichotomous variable to create the composite score. We also treated each element of sleep health (duration, timing, etc.) as having the same importance in our composite score, which could be inaccurate. In addition, our study had a large amount of missing data. Our sensitivity analysis demonstrated that using multiple imputations for missing data did not significantly impact our results. Last, the high prevalence of “good” sleep health in our sample and lack of variability in this variable likely decreased our ability to see large differences in outcomes between children with high vs. low sleep health.

Despite these limitations, we believe that our large sample size and diverse population make this study valuable as a high-powered assessment of children’s sleep health and cognition. This study prompts important questions about the effects of children’s sleep on cognition and offers evidence that disputes the notion that children’s cognitive functioning is associated with sleep. Further, in the future, with repeated measures, ECHO data may be able to be analyzed again to add more temporally sequenced repeated assessments of both sleep health and cognitive functioning that would add the strength of a cohort analysis over time.

## Conclusions

Contrary to our hypotheses, we found that a novel measure of sleep health in middle childhood was not associated with inhibitory control and other domains of cognitive functioning when administered cross-sectionally. It is plausible that the cumulative effects of poor sleep health over time are more robustly predictive of cognitive deficits than simply measuring sleep health at a singular time point. Future research could benefit from measuring the cumulative impact of sleep health on cognitive functioning over time. The finding that most children in our sample had good sleep health is also encouraging, as it suggests that many children are meeting recommended sleep guidelines (although this varied considerably across domains: 58 per cent for latency and 90 per cent for alertness). Future research could examine whether this finding generalizes to the US population.

## Supplementary Material

EC0667_Supplemental_Materials_Sleep_Advances_RR2_5_14_25_zpaf049

## Data Availability

Select de-identified data from the ECHO Program are available through NICHD’s Data and Specimen Hub (DASH). Information on study data not available on DASH, such as some Indigenous datasets, can be found on the ECHO study DASH webpage.
