# Supplementary material for: Associations between a novel measure of sleep health and cognitive functioning in middle childhood: a crosssectional Environmental Influences on Child Health Outcomes cohort study
Source: Sleep Adv. 2025 Aug 18;6(3):zpaf049. doi: 10.1093/sleepadvances/zpaf049 (PMC12413863; doi:10.1093/sleepadvances/zpaf049)
Supplement: EC0667_Supplemental_Materials_Sleep_Advances_RR2_5_14_25_zpaf049 [file ec0667_supplemental_materials_sleep_advances_rr2_5_14_25_zpaf049.docx]

**Supplemental Materials**

**Table S1.** Generalized estimating equations results evaluating the associations between self-reported sleep health and cognitive variables without imputed data.

**Table S2.** Generalized estimating equations results evaluating the associations between parent-reported sleep health and cognitive variables without imputed data.

**Figure S1.** Random forest model of the goodness of fit of the parent-report data.

**Figure S2.** Random forest model of the goodness of fit of the self-report data.

**Table S1.** Generalized estimating equations results evaluating the associations between self-reported sleep health and cognitive variables without imputed data.

| **Primary Outcomes** | **Unadjusted**  **coefficient** |  | **SE** | **95% CI**  **LL** | **95% CI**  **UL** | **p** |
| --- | --- | --- | --- | --- | --- | --- |
| Sleep health composite and inhibitory control | -0.52 |  | 0.39 | -1.13 | -0.02 | 0.04 |
| Sleep health composite and working memory | 0.26 |  | 0.44 | -0.49 | 1.11 | 0.81 |
| Sleep health composite and processing speed | -0.12 |  | 0.82 | -1.24 | 1.01 | 0.46 |
| Sleep health composite and cognitive flexibility | 0.14 |  | 0.54 | -0.89 | 1.1 | 0.98 |
| Sleep health composite and episodic memory | 0.14 |  | 0.51 | -0.84 | 1.02 | 0.71 |
| Sleep health composite and reading decoding | 0.54 |  | 0.65 | -0.57 | 1.71 | 0.45 |
| Sleep health composite and receptive vocabulary | 0.33 |  | 0.32 | -0.51 | 1.12 | 0.38 |

CI indicates confidence interval; LL, lower limit; SE, standard error; UL, upper limit.

**Table S2.** Generalized estimating equations results evaluating the associations between parent-reported sleep health and cognitive variables without imputed data.

| **Primary Outcomes** | **Unadjusted**  **coefficient** | **SE** | **95% CI**  **LL** | **95% CI**  **UL** | **p** |
| --- | --- | --- | --- | --- | --- |
| Sleep health composite and  inhibitory control | 0.37 | 0.76 | -.016 | 0.89 | 0.26 |
| Sleep health composite and  working memory | 0.15 | 0.42 | -0.68 | 1.14 | 0.76 |
| Sleep health composite and  processing speed | 0.62 | 0.78 | -1.21 | 2.62 | 0.58 |
| Sleep health composite and  cognitive flexibility | 0.63 | 0.53 | -0.46 | 1.65 | 0.49 |
| Sleep health composite and  episodic memory | 0.87 | 0.44 | 0.11 | 1.74 | 0.07 |
| Sleep health composite and  reading decoding | 0.4 | 0.46 | -0.32 | 1.18 | 0.53 |
| Sleep health composite and  receptive vocabulary | 0.65 | 0.32 | 0.04 | 1.36 | 0.05 |

CI indicates confidence interval; LL, lower limit; SE, standard error; UL, upper limit.

**Figure S1.** Random forest model of the goodness of fit of the parent-report data.


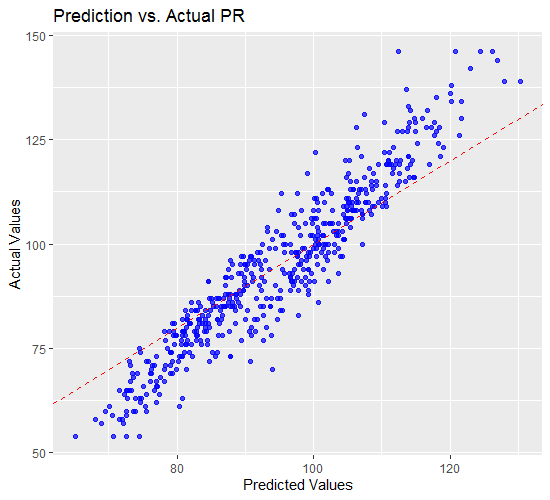


PR indicates parent report.

**Figure S2.** Random forest model of the goodness of fit of the self-report data.

**
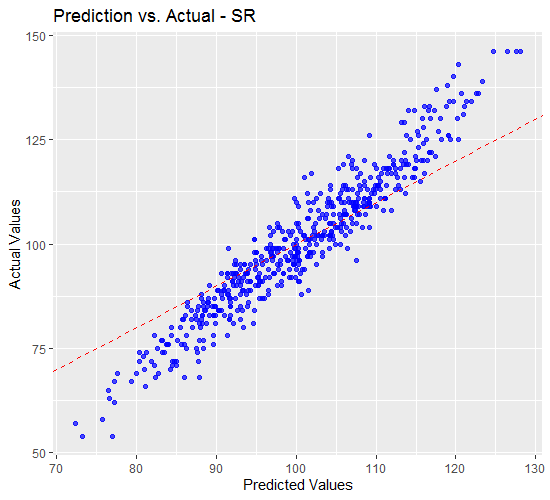
**

SR indicates self-report.
